# Molecular mechanistic insight into the anti-hyperuricemic effect of *Eucommia ulmoides* in mice and rats

**DOI:** 10.1080/13880209.2019.1568510

**Published:** 2019-03-07

**Authors:** Cong Fang, Lanying Chen, Mingzhen He, Yingying Luo, Mengjing Zhou, Ni Zhang, Jinfeng Yuan, Huiling Wang, Yongyan Xie

**Affiliations:** a National Pharmaceutical Engineering Center for Solid Preparation in Chinese Herbal Medicine, Jiangxi University of Traditional Chinese Medicine, Nanchang, China;; b School of Basic Medicine, Jiangxi University of Traditional Chinese Medicine, Nanchang, China

**Keywords:** Organic anion transporter 1, organic anion transporter 3, glucose transporter 9, uric acid transporter 1

## Abstract

**Context:***Eucommia ulmoides* Oliver (Eucommiaceae) has various medicinal properties. Our previous studies revealed that *Eucommia ulmoides* has a protective effect on hyperuricaemia.

**Objective:** This study investigates the effect of *Eucommia ulmoides* cortex ethanol extract (EU) on hyperuricaemia and explores the underlying mechanism in Kunming mice and Sprague–Dawley rats.

**Material and methods:** Sixty mice and sixty rats were divided into normal control, hyperuricaemia, allopurinol (10 mg/kg) and three EU groups. The EU groups received intragastric EU at 80, 160, 320 mg/kg in mice and 100, 200, 400 mg/kg in rats for 7 days. Serum uric acid (SUA) was measured using a kit. mRNA and proteins were quantified by RT-qPCR and immunohistochemical assays (IHC), respectively.

**Results:** The Maximal Tolerable Dose (MTD) of EU administered intragastrically was 18 g/kg in mice. The intermediate (160 mg/kg) and high (320 mg/kg) EU treatment significantly reduced (*p* < 0.05) SUA levels to 130.16 μmol/L and 109.29 μmol/L, respectively, and markedly elevated the mRNA expression of organic anion transporters 1 (OAT1) and organic anion transporters 3 (OAT3), while significantly deceasing the mRNA levels of glucose transporter 9 (GLUT9) and uric acid transporter 1 (URAT1) in the mouse kidney (*p* < 0.05). In hyperuricemic rats, high EU (400 mg/kg) significantly reduced SUA levels to 253.85 μmol/L, and increased OAT1 and OAT3 levels, but decreased URAT1 and GLUT9, compared to the hyperuricaemia group (*p* < 0.05).

**Discussion and conclusions:** This study demonstrated the potential hyperuricaemia ameliorating effect of EU. Specific active ingredients in EU should be evaluated. These results are valuable for the development of antihyperuritic agents from EU.

## Introduction

The incidence of hyperuricaemia is increasing worldwide (Johnson et al. 2013). Hyperuricaemia presents as a metabolic disturbance, which is closely correlated with gout, kidney disease, diabetes, hypertension, heart disease, and metabolic syndrome (Becker and Jolly 2006; So and Thorens 2010). Drug treatment of hyperuricaemia is associated with some adverse reactions. For example, allopurinol, benzbromarone and febuxostat could cause renal impairment, urinary calculi, and other adverse reactions (Wortmann 2002). Hence, it is necessary to explore novel and safe agents to control uric acid levels.

*Eucommia ulmoides* Oliver (Eucommiaceae), a Chinese traditional medicine, is used as a tonic in China, Japan, Korea, and other countries (He et al. 2014). *E. ulmoides* contains enriched chemical components such as lignans, iridoids, phenylpropanoids, flavonoids, and phenol, which possess various medicinal properties (Hussain et al. 2016; Yan et al. 2018). It has been used as a functional food to strengthen muscles, improve liver and kidney function, and increase life expectancy (Yen and Hsieh 2000). *E. ulmoides* also has a protective effect on renal function in mice and rats (Niu et al. 2016; Do et al. 2018). Geniposidic acid and chlorogenic acid are used to authenticate *E. ulmoides*, according to the Chinese Pharmacopoeia. Geniposidic acid, chlorogenic acid, geniposide, pinoresinol diglucoside, rutin, and quercetin are the primary components of *E. ulmoides* (He et al. 2014). Previous studies have demonstrated that chlorogenic acid, rutin, and quercetin exert an anti-hyperuricaemia effect in hyperuricemic mice (Hu et al. 2012; Chen et al. 2013; Meng et al. 2014; Xie et al. 2015; Ferraz-Filha et al. 2017). Therefore, we hypothesized that it may have a protective effect on hyperuricaemia.

This study investigates whether EU has an effect on hyperuricaemia and explores its underlying molecular mechanisms.

## Materials and methods

### Chemicals and reagents

Hypoxanthine (HX, 99%) and oxonic acid potassium salt (OAS, 97%) were obtained from Sigma-Aldrich Co. LLC (USA). Allopurinol was purchased from Shanghai Xinyi Wanxiang Pharmaceutical Group Ltd. (Shanghai, China). UA kit was purchased from Nanjing Jian Cheng Bioengineering Institute (Nanjing, China). SP Rabbit HRP (DAB) immunohistochemical kit was obtained from Jiangsu Kang Wei Shi Ji Biotechnology Co. Ltd. (Jiangsu, China). The primers were purchased from Genscript (Nanjing, China), Trizol was commercially obtained from Invitrogen (USA), and Revert Aid First Strand cDNA Synthesis Kit and PowerUp™ SYBR™ Green Master Mix were obtained from Thermo Fisher Scientific Inc. (USA). Anti-OAT1 was purchased from Abcam Inc. (Cambridge, UK), Anti-OAT3 from Changzhou Xiangtai Biotechnology Co. Ltd. (Changzhou, China), Anti-URAT1 from Abbiotec Inc. (San Diego, CA, USA). Anti-GLUT9 was purchased from Millipore Inc. (USA). Other biochemical reagents and chemicals were of analytical grade.

### Plant material and extraction

The cortex of *E. ulmoides* (batch number: 1706005) was purchased from Tian Qi Tang pharmacy (Zhangshu China) and identified by Bei Wang, the Vice Director of Pharmacists from the Nanchang Institute for Drug Control (Nanchang, China). Voucher specimens were deposited at the laboratory of the Jiangxi University of Traditional Chinese Medicine. The cortex of *E. ulmoides* (505.73 g) was washed, powdered and then extracted twice with 70% ethanol (1:8 w-v, 1.5 h each time). Then, the extract was evaporated *in vacuo* to yield a 70% ethanol extract (EU) (72.53 g; 14.34%) (Wang et al. 2016).

### Chromatographic analysis conditions

The EU sample was analyzed using a Waters SYNAPT G2-Si QTOF LC/MS system (Waters MS Technologies, UK) for phytochemical characterization. A Welch Ultimate XB-C18 column (Welch Materials, Inc. Shanghai, China, 2.1 mm × 100 mm, 1.7 µm) was utilized for analysis with a flow rate of 0.35 mL/min. The mobile phase consisted of acetonitrile (solvent B) and 0.1% formic acid aqueous solution (solvent A), using a linear gradient elution: 5–13% B (0–5 min); 13–18% B (5–10 min); 18–60% B (10–35 min), Negative ionization mode was used in all acquisitions. In addition, we recorded the range *m/z* = 50–1000 with mass-measurement of all peaks in the mass spectra.

### Animals experiment

Eighty male and 20 female Kunming mice (18 ± 2 g) were purchased from Beijing Vital River Laboratory Animal Technology Co., Ltd. China (Certificate No. SCXK 2016-0011). Sixty male SD rats (180 ± 20 g) were purchased from Hunan SJA Laboratory Animal Co., Ltd., China (Certificate No. SCXK 2016-0002). The animals were housed in cages and maintained (22 ± 1 °C and 40–50% humidity) under 12 h light/dark cycle and provided standard laboratory food and water *ad libitum*. All the animals were maintained under the above conditions for 7 days before the start of the experiment for acclimatization. The experimental procedures were reviewed and approved by the Institutional Animal Care and Use Committee of the Jiangxi University of Traditional Chinese Medicine. In the acute oral toxicity study, mice were divided into two groups (EU and control; *n* = 10/sex/group), The EU group was orally treated with maximal dosage of EU (18 g/kg). The control group received the vehicle. General behaviour and mortality were observed for up to 14 days. All protocols were in accordance with the traditional Chinese medicine and natural medicine acute toxicity study guidelines. In the anti-hyperuricaemic experiment, mice and rats were divided into normal control (*n* = 10), hyperuricaemia (*n* = 10), allopurinol (*n* = 10), and EU at low (*n* = 10), intermediate (*n* = 10), and high (*n* = 10) groups. Hyperuricaemia was induced in the hyperuricaemia, allopurinol, and the three EU groups both in mice and rats. In mice, hyperuricaemia was induced by intraperitoneal injection of oxonic acid potassium salt (300 mg/kg) for 7 consecutive days, and in the rats, hyperuricaemia was induced by intragastric administration of hypoxanthine (200 mg/kg) followed by intraperitoneal injection of oxonic acid potassium salt (200 mg/kg) 1 h later. The allopurinol group in both mice and rats received intragastric allopurinol (10 mg/kg) for 7 days. The three mouse EU groups received intragastric EU 80 mg/kg (low EU), 160 mg/kg (intermediate EU), or 320 mg/kg (high EU) for 7 days and the three rat EU groups received intragastric EU 100 mg/kg (low EU), 200 mg/kg (intermediate EU), or 400 mg/kg (high EU) for 7 days. Mouse blood samples were collected 1 h after injections, while rat blood samples were collected 3 h after injections. The animals were euthanatized, after the 7 day treatment period and the kidney and liver were collected.

### RT-qPCR

Total RNA from the renal tissue samples was extracted by Trizol, followed by cDNA synthesis using the Revert Aid First Strand cDNA Synthesis Kit (Eppendorf Mastercyclear). The primers are listed in Table 1. The PCR conditions were as follows: pre-denaturation at 95 °C for 1 min, 40 cycles at 95 °C for 3 s, and 60 °C for 20 s. The quantity of mRNA was calculated through the cycle threshold (CT) values, which was calculated using the software v.2.0.6 (Applied Biosystems 7500). The relative mRNA expression levels were determined using the 2^–ΔΔCt^ method.

**Table 1.** List of primer sequences for RT-qPCR.

Gene	Forward primer	Reverse primer RefSeq
OAT1	5′-GAGCAGAGGAAAGCAGAAGC-3′	5′-CCCTTTAGTGCTGTGTGACG-3′ NM_008766.3
OAT3	5′-TACAGTTGTCCGTGTCTGCT-3′	5′-CTTCCTCCTTCTTGCCGTTG-3′ NM_031194.5
URAT1	5′-AGGTCCTGACAGGTTCTGT-3′	5′-CTCTGCCTTCCTCCTGTTGA-3′ NM_009203.3
GLUT9	5′-TTCGGGTCCTCCTTCCTCTA-3′	5′-GGACACAGTCACAGACCAGA-3′ NM_001102414.1
GAPDH	5′-GGCACAGTCAAGGCTGGAATG-3′	5′-ATGGTGGTGAAGACGCCAGTA-3′ NM_008084.3

### Immunohistochemistry

Paraffin-embedded renal slices (3 μm) were used for immunohistochemical analysis. In brief, the slices were stained following according to the manufacturer’s instructions (Jiangsu Kang Wei Shi Ji Biotechnology Co. Ltd.). Slices were incubated with primary antibodies OAT1 (1:400), OAT3 (1:100), URAT1 (1:200) and GLUT9 (1:2000) overnight at 4 °C. The results of immunohistochemical staining are presented as the mean integrated optical density and were analyzed using Imagine Pro-Plus 6.0 software.

### Statistical analysis

Statistical analysis was performed using one-way analysis of variance (ANOVA), followed by multiple comparisons using Dunn’s test; *t*-test was used for statistical analyses of two independent groups. All tests were performed using SPSS 19 and a *p* value < 0.05 was considered as statistically significant.

## Results

### Acute toxicity study

**Figure 1.**
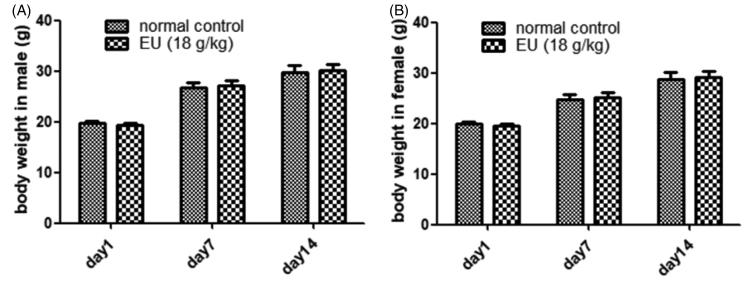
Effects of oral EU on body weight (g). (A) Male mice treated with EU in acute toxicity test. Data are means ± SD (*N* = 10). (B) Female mice treated with EU in acute toxicity test. Data are means ± SD (*N* = 10).

The acute toxicity study showed no significant changes in body weight (Figure 1(A,B)), respiration, reflex, behaviour changes, or death, indicating the MTD for oral administration of EU as 18 g/kg.

### Analysis of EU and identification of its major components by using HPLC/MS

**Figure 2.**
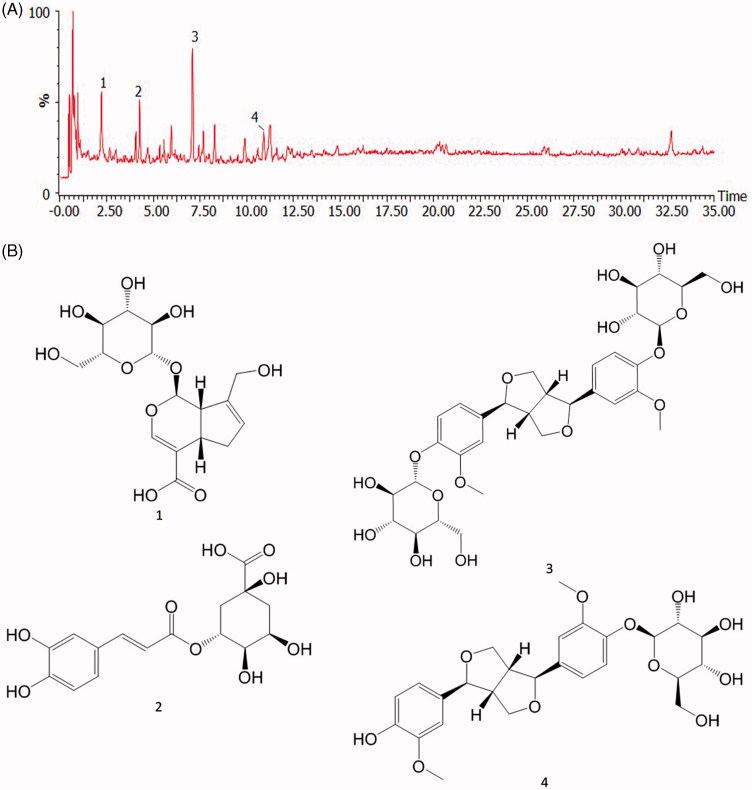
HPLC Chromatogram of EU (A) and chemical structures of major components (B). Analysis of EU extract was performed by using Q-TOF -LC/MS. 1: geniposidic acid, 2: chlorogenic acid, 3: Pinoresinol Diglucoside, 4: (+) piresil-4-*O*-β-D-glucopyraside.

An optimized chromatographic condition to determine the major constituents of EU was designed. The mobile phase consisting of acetonitrile 0.1% formic acid aqueous solution was used for the analysis of EU. The presence of four compounds, geniposidic acid **(1)** (*m/z* 373.1297 at tR 2.2 min), chlorogenic acid **(2)** (*m/z* 353.0872 at tR 4.3 min), pinoresinol diglucoside **(3)** (*m/z* 681.2634 at tR 7.1 min), and (+) piresil-4-*O*-β-d-glucopyraside **(4)** (*m/z* 519.2110 at tR 10.9 min) in EU was defined by comparing the retention time, precise molecular mass, and MS/MS fragmentations with standard compounds (Figure 2(A,B)).

### Effect of EU on SUA levels in hyperuricaemia mice

**Figure 3.**
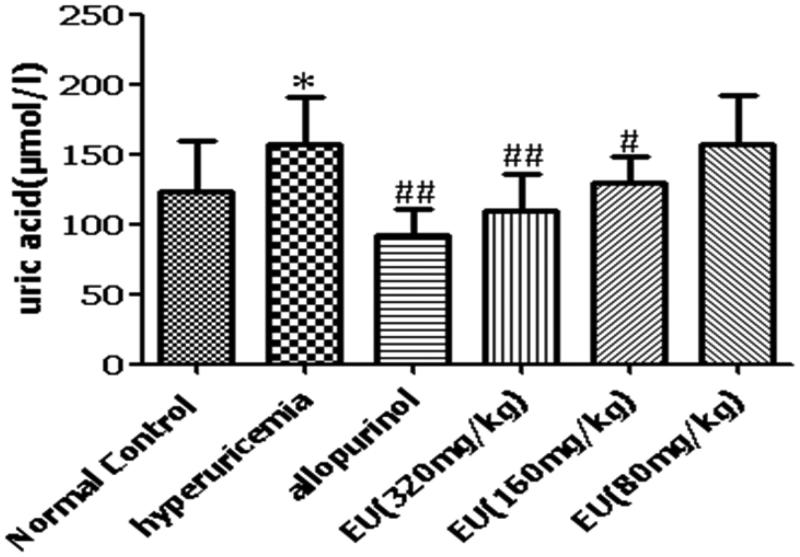
Effect of EU on SUA levels in hyperuricemia mice. *p < 0.05, compared to the normal control; #*p* < 0.05, ##*p* < 0.01 as compared to the hyperuricemia group.

The serum uric acid (SUA) levels of the hyperuricaemia group were significantly increased compared to the normal control (*p* < 0.05). Compared with the hyperuricaemia group, the SUA levels of allopurinol group was decreased significantly (*p* < 0.01). There was a significant decrease in SUA levels (*p* < 0.05) in the high (320 mg/kg) and intermediate (160 mg/kg) groups (Figure 3).

### Effects of EU on the mRNA expression of OAT1, OAT3, URAT1 and GLUT9 in hyperuricaemia mice

**Figure 4.**
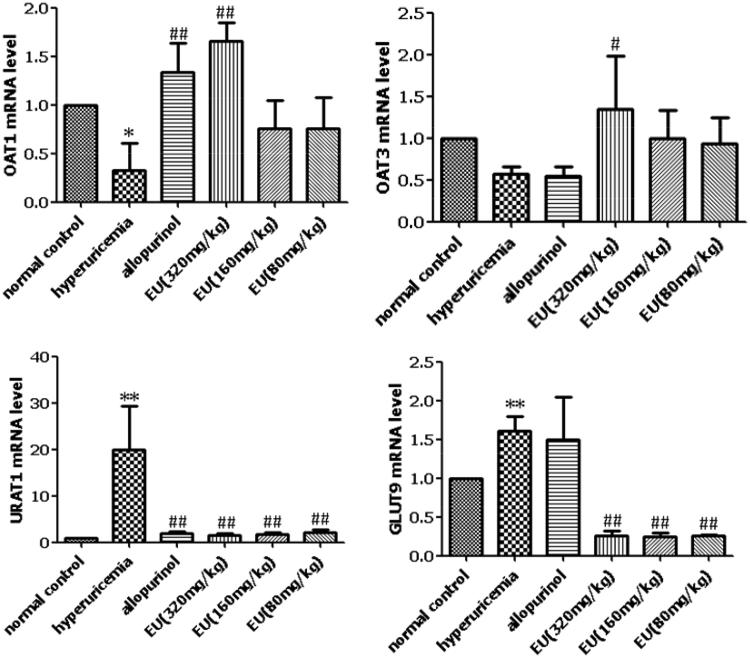
Effects of EU on the mRNA expression of OAT1, OAT3, URAT1 and GLUT9 in hyperuricemia mice. *p < 0.05,**p < 0.01 as compared to the normal control; #*p* < 0.05, ##*p* < 0.01 as compared to the hyperuricemia group.

Compared to the normal controls, the levels of renal mURAT1 and mGLUT9 were significantly increased in the hyperuricaemia group. The levels of mOAT1 and mOAT3 were significantly lower in the hyperuricaemia group, compared to the normal controls. The high EU group showed significantly increased expressions of mOAT1 and mOAT3, compared to the hyperuricaemia group, and all the EU groups (low, intermediate, and high) showed significantly decreased expression of mURAT1 and mGLUT9 (Figure 4).

### Effect of EU on SUA levels in hyperuricaemia rats

**Figure 5.**
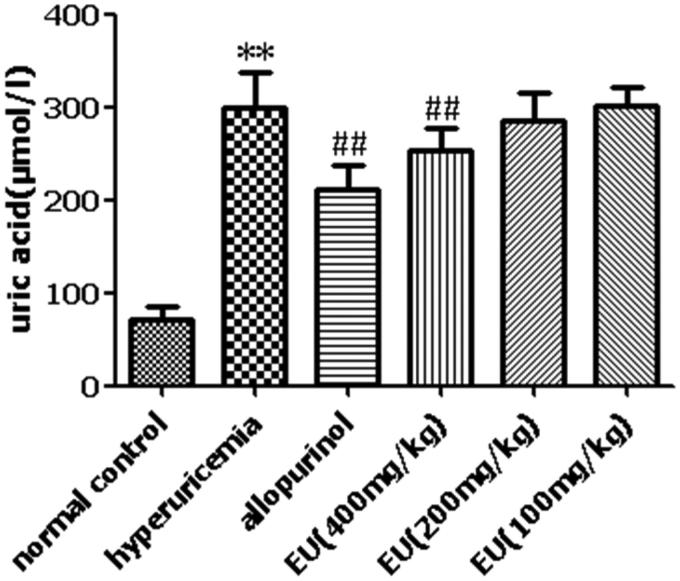
Effect of EU on SUA levels in hyperuricemia rats. **p < 0.01 as compared to the normal control; ##*p* < 0.01 as compared to the hyperuricemia group.

The SUA levels in the hyperuricaemia group were significantly increased compared to the normal controls (*p* < 0.05). The allopurinol group had significantly lower SUA (*p* < 0.01) compared to the hyperuricaemia group. There was a marked reduction in SUA levels (*p* < 0.05) in the high EU group (Figure 5).

### Effect of EU on the expressions of OAT1, OAT3, URAT1 and GLUT9 in hyperuricaemia rats

**Figure 7.**
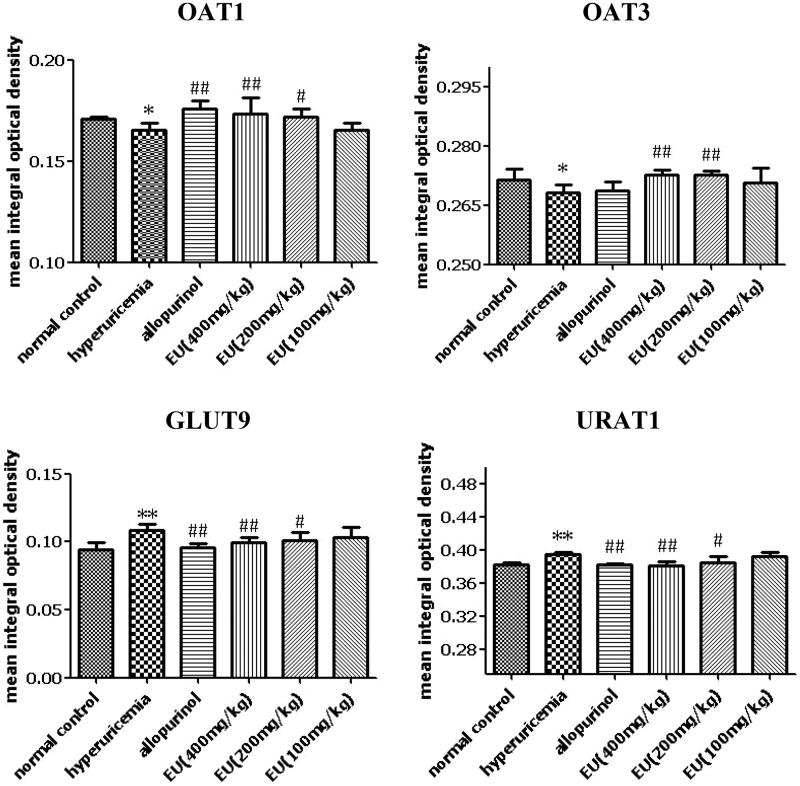
Effect of EU on the expression of OAT1, OAT3, URAT1 and GLUT9 in hyperuricemia rats. *p < 0.05, **p < 0.01 as compared to the normal control; #*p* < 0.05, ##*p* < 0.01 as compared to the hyperuricemia group.

As shown in Figures 6 and 7, the expression of OAT1, OAT3, URAT1 and GLUT9 could be detected in the renal tissue of rats in normal control. Compared to the normal controls, the levels of URAT1 and GLUT9 were significantly increased in the hyperuricaemia group. The expressions of URAT1 and GLUT9 in intermediate and high EU groups were significantly decreased in comparison to the hyperuricaemia group (*p* < 0.01). While the expressions of OAT1 and OAT3 were markedly reduced in the hyperuricaemia group compared to the normal control, and intermediate and high EU treatment resulted in the upregulated expressions of OAT1 and OAT3 compared to the hyperuricaemia group (*p* < 0.01).

Figure 6.Effect of EU on the expression of OAT1, OAT3, URAT1 and GLUT9 in hyperuricemia rats (200x).

**Figure:**
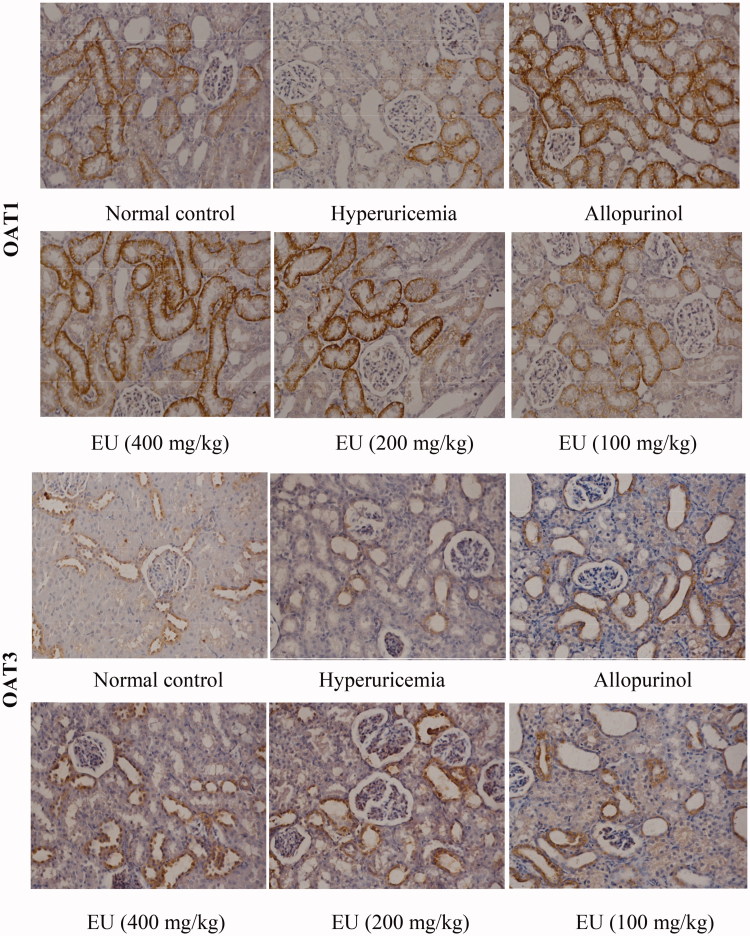

**Figure:**
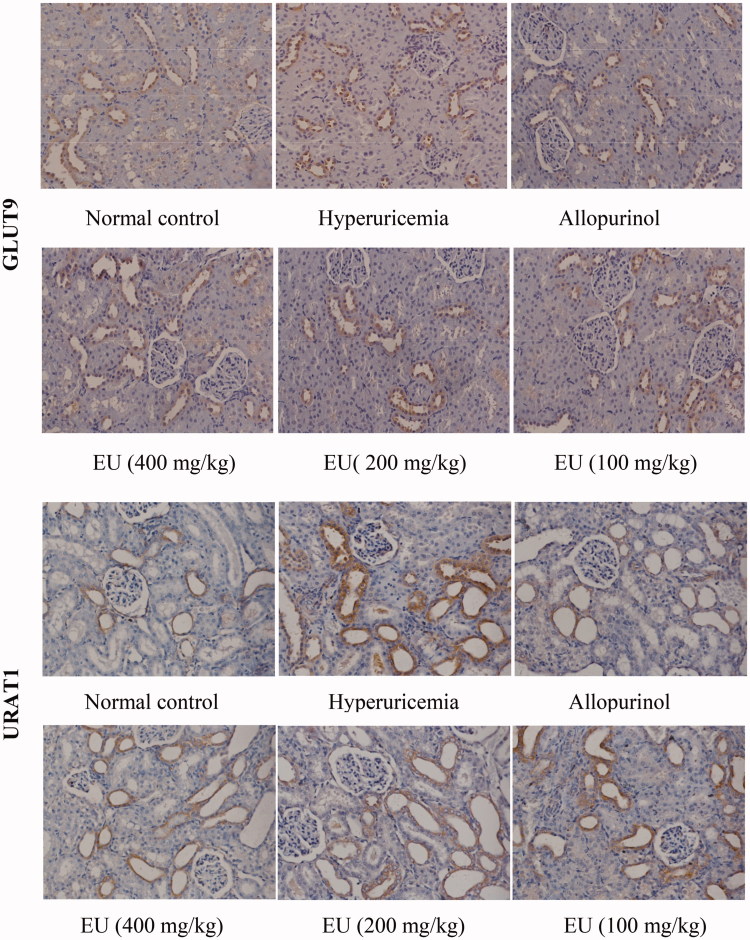

## Discussion

In the present study, the effects and possible mechanisms of EU on hyperuricemic mice and rats were investigated. EU was likely not toxic, and the anti-hyperuricemic activity of EU was related to upregulation of OAT1 and OAT3, down-regulation of URAT1 and GLUT9 in the kidney.

High uric acid levels are an important risk factor for kidney disease, cardiovascular disease, ischaemic stroke and other diseases (Chen et al. 2009; Storhaug et al. 2013). Allopurinol is one of the most frequent drugs used to reduce uric acid. Nevertheless, the development of adverse effects side contributes to the abandoned treatment discontinuation in many patients (Umamaheswari et al. 2009). Hence, it is necessary to find a better antihyperuricemic drugs. This study showed that treatment with EU (320, 160 mg/kg) in hyperuricaemia mice and EU (400 mg/kg) in hyperuricaemia rats significantly decreased the serum uric acid levels.

Oxonic acid potassium salt and hypoxanthine are frequently employed to establish hyperuricaemia in animals. Hypoxanthine is a precursor of uric acid in the metabolism of uric acid (Kooij 1994) and oxonic acid potassium salt as an uricase inhibitor is used to induce hyperuricaemia in animal model (Yan et al. 2008; Hou et al. 2014). In the present study, oxonic acid potassium salt was used to induce hyperuricaemia in mice, and a combination of oxonic acid potassium salt and hypoxanthine was utilized to induce hyperuricaemia in rats (Hou et al. 2012; Yong et al. 2016; Zhang et al. 2017).

The kidney is the main organ responsible for uric acid excretion. Uric acid is excreted by the kidney through four processes: glomerular filtration, reabsorption in proximal tubules, secretion and reabsorption after secretion (Richette and Bardin 2010). It has been reported that hyperuricaemia can be caused by the reduced excretion/secretion of urate (Habu et al. 2003; Boffetta et al. 2009). Hyperuricaemia is induced by dysfunctional urate transporters OAT1, OAT3, GLUT9, and URAT1 are dysfunctional, hyperuricaemia will be induced (Habu et al. 2003; Enomoto and Endou 2005; Eraly et al. 2008; Preitner et al. 2009). Uric acid transporters play an important role in transporting uric acid in the renal tubule. OAT1 and OAT3 are located at the basolateral membrane, which regulates the entry of uric acid into the proximal tubule cells from the blood through the basolateral membrane (Bobulescu and Moe 2012). A previous study found that in mice with OAT1 gene knockout, the ability of the renal tubules to secrete uric acid salt was significantly weakened (Eraly et al. 2008). It is reported that OAT3 excreted uric acid through exchange of organic ions and dicarboxylic acid, which is similar to the function of OAT1 (Bakhiya et al. 2003). In this study, EU treatment significantly increased the expression of mOAT1 and mOAT3 in mice and the protein expression of OAT1 and OAT3 in rats. URAT1, encoded by SLC22A12, is a protein and is distributed in the brush border, membrane of the renal tubular epithelial cells responsible for uric acid reabsorption, and is a risk factor for hyperuricaemia. GLUT9, encoded by SLC2A9, is a novel target for hyperuricaemia. SLC2A9 splice variant acts as a high-capacity urate transporter (Wu et al. 2014). Studies have shown that the mutation of SLC2A9 gene leads to renal hypouricaemia, which may be related to the decrease of the reabsorption function caused by the deficiency of GLUT9 (Matsuo et al. 2008). In the present study, EU treatment significantly decreased the expression of mURAT1 and mGLUT9 in mice and the protein expression of URAT1 and GLUT9 in rats. Our results show that EU regulates SUA levels by enhancing uric acid excretion and inhibiting uric acid reabsorption in the renal tubules. Our results also revealed the underlying mechanism by EU exerts this beneficial role. We demonstrate that EU treatment up-regulates the levels of OAT1 and OAT3, and down-regulates the renal levels of URAT1 and GLUT9 in the kidney.

Xanthine oxidase (XOD) is widely found in tissues, especially in the liver. XOD is an important enzyme in the purine metabolic pathway, which is responsible for catalyzing the oxidation of hypoxanthine to xanthine, and then to uric acid (UA) (Stabler et al. 2015). To explore the relevant mechanisms, we also assessed the impact of EU on XOD activity. However, we found that EU had no effect on XOD levels in the serum and liver.

As far as we know, this is the first report describing the anti-hyperuricemic effect of EU regulates renal OAT1, OAT3, URAT1 and GLUT9, and enhances uric acid excretion and inhibits uric acid reabsorption in the renal tubules. However, the specific active ingredients in EU and its effect in other animals should be studied in detail.

## Conclusions

In this study, EU was likely not toxic and exerted its anti-hyperuricaemic effects by increasing the expression of OAT1 and OAT3, as well as decreasing the expression of URAT1 and GLUT9. However, further studies are needed and the effect in other animals remains to be verified. Based on these findings, EU can be used as an effective substance in the development of drugs for hyperuricaemia.
